# Improvement of antibody affinity by introduction of basic amino acid residues into the framework region

**DOI:** 10.1016/j.bbrep.2018.07.005

**Published:** 2018-07-14

**Authors:** Atsushi Fukunaga, Shingo Maeta, Bajaj Reema, Makoto Nakakido, Kouhei Tsumoto

**Affiliations:** a Technology Development, Sysmex Corporation, 4-4-4 Takatsukadai, Nishi-ku, Kobe, Japan; b Medical Proteomics Laboratory, Institute of Medical Science, University of Tokyo, Minato-ku, 108-8639 Tokyo, Japan; c Department of Chemistry and Biotechnology and University of Tokyo, Bunkyo-ku, Tokyo 103-0081, Japan.; d Department of Bioengineering, School of Engineering, University of Tokyo, Bunkyo-ku, Tokyo 103-0081, Japan

**Keywords:** Fab, Charged amino acid residue, SPR, Encounter complex

## Abstract

Antibodies are widely used not only as therapeutic agents but also as research tools and diagnostic agents, and extensive efforts have been made to generate antibodies that have higher affinity. It was recently reported that introduction of charged residues into the framework region of an antibody improved its affinity; however, the underlying molecular mechanism has not been elucidated. In this study, we used kinetic and thermodynamic analyses of the antibody–antigen interaction to investigate the molecular mechanism by which an antibody with introduced charged residues recognizes its antigen with higher affinity. The introduction of basic amino acid residues resulted in improvement of the affinity whereas the introduction of acidic residues weakened the interaction. For two mutant antigen-binding fragments (Fabs) with improved affinity (named K5- and R5-mutants), the balance between the association rate constant *k*_on_ and the dissociation rate constant *k*_off_ was distinct despite each mutant having the same number of charged residues. Moreover, thermodynamic analysis of the interactions in the transition state revealed a difference between the K5- and R5-mutants in terms of enthalpic energy change following formation of the encounter complex with the antigen. These results suggest that the affinity of the K5- and R5-mutants is improved by distinct mechanisms. Although the mutations destabilize the Fab and necessitate further studies, our strategy is expected to become a versatile and simple means to improve the affinity of antibodies to their antigens.

## Highlights

• Introduction of charged residues into framework improved the affinity to its antigen.
• Kinetic parameters were distinct despite the same number of basic charged residues.
• The introduction of basic residues would affect the encounter complex formation.

## Introduction

Antibodies are widely used not only as therapeutic agents but also as research tools and diagnostic agents due to their high specificity and affinity towards their antigens [1]. Antibodies acquire their affinity and specificity towards a variety of target antigens by changing the composition of amino acid residues in the six hyper-variable regions known as complementarity-determining regions (CDRs)[2]. Although CDRs comprise only a small number of amino acid residues, antibodies can precisely recognize numerous types of antigen [3]. As the high affinity of an antibody towards its antigen is a critical factor for therapeutic applications such as molecularly targeted anti-cancer drugs [4], [5], [6], [7], extensive efforts have been made to generate higher-affinity antibodies, mainly through a directed evolutionary approach [8], [9], [10], [11], [12].

Recently it was reported that the affinity of an antibody towards its antigen was improved by introducing charged amino acid residues into the framework region of the antibody [13]; however, the molecular mechanism by which the modified antibody recognized its antigen with higher affinity remained to be elucidated. Here, we investigated the molecular mechanism by which introduction of charged amino acid residues affects an antibody's recognition of the antigen. Although the affinity was improved by introducing basic amino acid residues (either arginine or lysine), the thermodynamic parameters of the antibody–antigen interaction in the transition state were significantly different. Our results suggested that the introduction of basic residues into the framework region of antibodies improved the affinity by distinct mechanisms. A more detailed characterization of the interaction between antigens and antibodies with charged residues would contribute to the development of a versatile strategy to improve the affinity of antibodies.

## Materials and methods

### Genetic engineering and protein expression and purification

We used an anti-insulin Fab, an antigen binding fragment of antibodies, as a model. The mouse monoclonal antibody against human insulin was developed by conventional hybridoma technology from Sysmex Corporation [14]. All mutants in framework region 3 (FR3) were generated by site-directed mutagenesis using a KOD -Plus- Mutagenesis Kit (Toyobo) in accordance with the manufacturer's protocol.

Antibodies were expressed using the pcDNA 3.4 TOPO expression vector (Life Technologies) and Expi293TM expression system (Life Technologies). Cell culture supernatant was filtered through a 0.8-μm-pore-size filter (ADVANTEC) and antibodies in the filtered supernatant were added to protein A resin (GE Healthcare). After washing with phosphate-buffered saline (PBS), antibodies were eluted with 0.1 M glycine-HCl (pH 2.7), neutralized with 100 mM Tris-HCl (pH 8.0), and dialyzed against PBS.

Fabs were prepared by using Mouse IgG1 Fab and F(ab′)2 Preparation Kits (Pierce). Eluted Fabs were further purified with size-exclusion chromatography using Superdex 200 Increase 10/300GL (GE Healthcare) in PBS.

### Binding and thermodynamic analysis of the interaction between Fabs and insulin using surface plasmon resonance (SPR)

To obtain kinetic and thermodynamic parameters, SPR experiments were performed using a BIAcoreT200 system (GE Healthcare). Insulin (Funakoshi) was immobilized on research-grade CM5 sensor chips (GE Healthcare). The amount of Insulin immobilized for kinetic analysis were determined according to the manufacture's protocol. Each purified Fab was dialyzed against HBS-EP buffer (10 mM HEPES [pH 7.4], 150 mM NaCl, 3.4 mM EDTA, 0.05% surfactant P20) and injected over the immobilized insulin at a flow rate of 50 µl/min. The data were normalized by subtracting the response from a blank cell in which bovine serum albumin (BSA) alone was immobilized. BIA evaluation software version 2.0.2.(GE Healthcare) was used to analyze the data. Kinetic parameters were calculated by a global fitting analysis with the assumptions of the 1:1 Langmuir binding model.

Thermodynamic analyses of each Fab were performed at five temperature points (283.15 K, 288.15 K, 293.15 K, 298.15 K, and 303.15 K). The standard state Gibbs energy change upon binding was obtained from Eq. (1):

(1) $$\Delta G=R\;T\;\ln\;K_{d}$$

where K_d_ is the dissociation constant, expressed in units of mol·l^−1^, R is the gas constant, and T is the absolute temperature. The ∆G values of each data set were plotted against the temperatures, and were fitted with the nonlinear van’t Hoff equation (Eq. (2)),

(2) ΔG=ΔH−TΔS+ΔCp(T−293.15)−ΔCpTln(T/293.15)

where ∆H and ∆S are the binding enthalpy change and entropy change at 293.15 K, respectively, and ∆Cp is the heat capacity change, which is assumed to be temperature independent.

The activation energy parameters were obtained from the temperature dependence of the association rate constant following the Eyring approximation:

$$\ln\left(k_{on}\right)/T)=-\left(\Delta H^{‡}/\mathrm{RT}\right)+\left(\Delta S^{‡}/R\right)+\;\ln\left(k_{B}/h\right)$$

where *k*_on_ is the association rate constant, ∆H^‡^ is the activation enthalpy, R is the gas constant, T is the absolute temperature, ∆S^‡^ is the activation entropy, *k*_*B*_ is the Boltzmann constant, and *h* is the Planck constant.

### Measurement of melting temperature (Tm) from differential scanning calorimetry (DSC)

The thermal stabilities of mutants were monitored with a VP-DSC MicroCalorimeter (Malvern). The concentrations of the proteins were 0.25 mg/ml in PBS (pH 7.4). Heating was at 1 °C/min, and the scanning was performed from 30 °C to 90 °C. The data were normalized by subtracting the response of PBS alone from the experimental responses measured.

## Results

### The effect of charged residue introduction on Fab binding

**Fig. 1:**
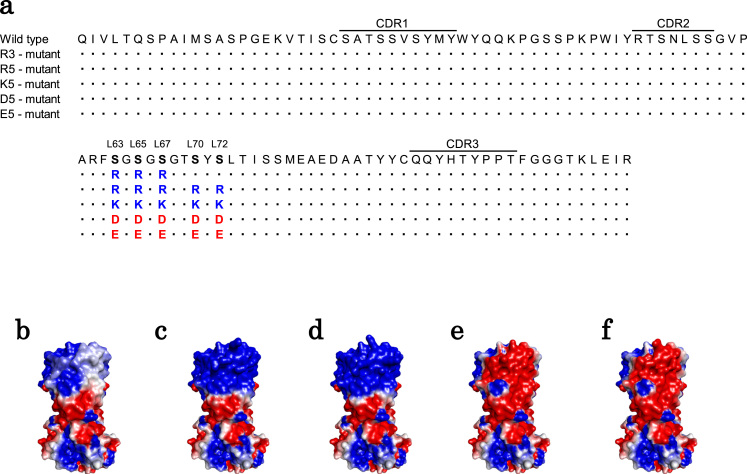
Light chain variable region (VL) amino acid sequences of mutants and surface representations of Fab mutants. (**a**) VL amino acid sequence of each mutant. L63, L65, L67, L70, and L72 were selected as mutation points. All points were included in framework region 3. The mutants were named R3-mutant, R5-mutant, K5-mutant, D5-mutant, and E5-mutant. (**b–f**) The electrostatic potentials around the VL binding site of wild type (b), R5-mutant (c), K5-mutant (d), D5-mutant (e), and E5-mutant (f) depicted by using Discovery Studio (ver. 4.5; BIOVIA) with contours drawn at 2 kT per electron at 0.018 mM NaCl (blue for positive and red for negative) by using only full charges. (For interpretation of the references to color in this figure legend, the reader is referred to the web version of this article)

We introduced three or five mutations into FR3 of the Fab following the previous study [13]; the mutants were named the R3-mutant (LS63R, LS65R, LS67R), R5-mutant (LS63R, LS65R, LS67R, LS70R, LS72R), K5-mutant (LS63K, LS65K, LS67K, LS70K, LS72K), D5-mutant (LS63D, LS65D, LS67D, LS70D, LS72D), and E5-mutant (LS63E, LS65E, LS67E, LS70E, LS72E) (Fig. 1a). The isoelectric point and electrostatic surface potential of each mutant were calculated by using Discovery Studio (ver. 4.5; BIOVIA). According to the calculation, the wild-type Fab has a weak positive charge in and around the insulin binding site, whereas the R5- and K5-mutants have a strong positive potential and the D5- and E5-mutants have a strong negative potential there (Fig. 1b–f). The change of the charge distribution may influence the electrostatic interaction.

**Fig. 2:**
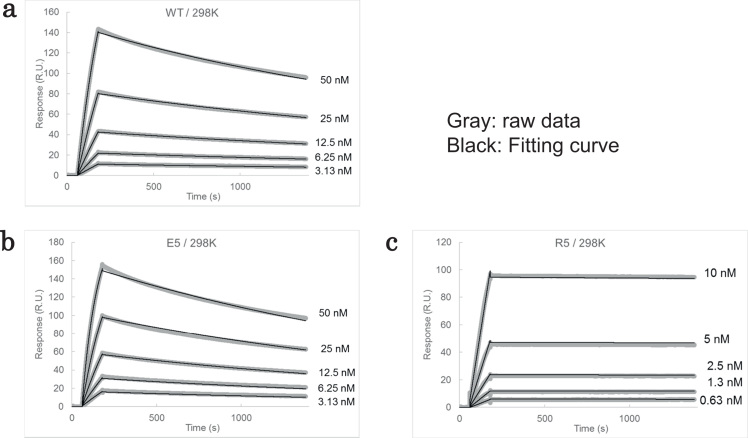
Kinetic analysis of the interaction between Fabs and insulin. SPR sensorgrams obtained with various Fabs are shown. All Fabs were at 5 nM. BIA evaluation software version 2.0.2 (GE Healthcare) was used to analyze the data.

To assess the effect of the mutations on the interaction between the Fab and insulin, we conducted SPR analysis and determined the kinetic parameters of the interactions for each mutant. The SPR sensorgrams for the experiments using wild-type or mutant Fab are shown in Fig. 2. The R5-mutant showed a notably slow dissociation compared to the wild-type Fab, whereas the response of the E5-mutant was smaller than that of the wild type. The kinetic parameters of the interactions at 25 °C for each mutant are summarized in Table 1. For the K5- and R5-mutants, the *k*_on_ values were, respectively, 22 and 2.9 times that of the wild type, whereas the *k*_on_ values of the D5- and E5-mutants were 0.31 and 0.11 times that of the wild type. The *k*_off_ values for all mutants except the R5-mutant were almost the same as that of the wild-type Fab, indicating that these mutations influenced only association. For the R5-mutant, the *k*_off_ value was 0.03 times that of the wild-type Fab. The R5-mutation was the only mutation that had an effect on both the association and the dissociation, suggesting that the improvement of the interaction by the R5-mutation was not brought about by simple electrostatic attraction of whole molecules.

**Table 1:** Kinetic parameters of Fab–insulin interactions at 25 °C.

**Analyte**	**K**_**d**_**(M)**	***k***_**on**_**(M**^**−1**^**s**^**−1**^**)**	***k***_**off**_**(s**^**−1**^**)**
Wild type	2.22 × 10^−10^	(2.48 ± 0.01) × 10^6^	(5.49 ± 0.01) × 10^−4^
R3-mutant	5.49 × 10^−11^	(8.79 ± 0.02) × 10^6^	(4.83 ± 0.01) × 10^−4^
R5-mutant	2.03 × 10^−12^	(7.19 ± 0.05) × 10^6^	(1.46 ± 0.01) × 10^−5^
K5-mutant	8.04 × 10^−12^	(5.44 ± 0.04) × 10^7^	(4.37 ± 0.01) × 10^−4^
D5-mutant	9.69 × 10^−10^	(7.64 ± 0.04) × 10^5^	(7.40 ± 0.02) × 10^−4^
E5-mutant	1.52 × 10^−9^	(2.79 ± 0.01) × 10^5^	(4.24 ± 0.01) × 10^−4^

The values obtained by global fitting are the means ± SE.

### Thermodynamic parameters for interactions in the steady and transition states

To gain an insight into the molecular mechanisms by which the mutations improve the affinity between the Fab and insulin, we conducted SPR analyses at different temperatures and evaluated the thermodynamic parameters of the interaction (Supplementary Fig. S1). For the wild-type and E5- and D5-mutants, energetically favorable entropic (ΔS) and unfavorable enthalpic (ΔH) changes were observed following the interaction. In contrast, the R3-, R5-, and K5-mutants each showed favorable enthalpic and entropic changes, suggesting that the favorable enthalpic energy would be derived from the electrostatic interaction. To examine the contribution of electrostatic charges on the affinity, we performed ELISA assay in the presence of different concentration of NaCl (Supplementary Fig. S2). The response gradually reduced with increasing concentration of NaCl, supporting the contribution of electrostatic charges on the interaction.

**Fig. 3:**
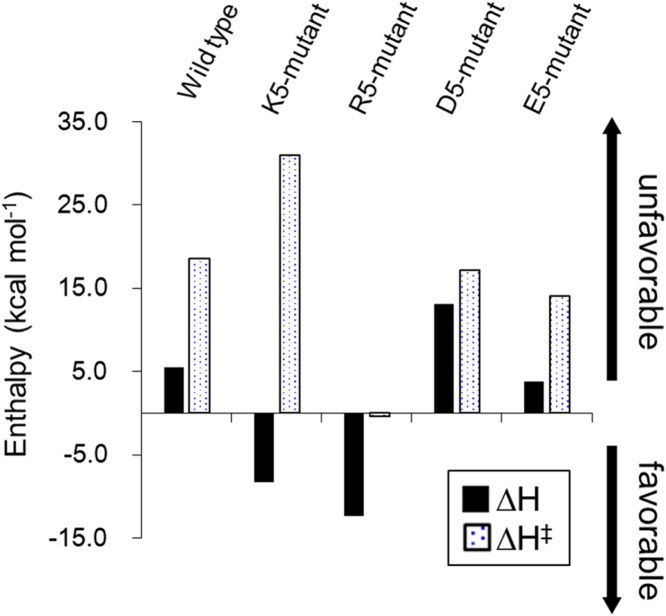
Analysis of the binding enthalpy. The activation energy parameters were obtained from the temperature dependence of the association rate constant following the Eyring approximation: ln (*k*_*on*_)/T) = −(∆H^‡^ / RT) + (∆S^‡^/R) + ln (*k*_B_*/h*) where *k*_*on*_ is the association rate constant, ∆H^‡^ is the activation enthalpy, R is the gas constant, T is the absolute temperature, ∆S^‡^ is the activation entropy, *k*_B_ is the Boltzmann constant, and *h* is the Planck constant.

Subsequently, we investigated the change in enthalpic energy in the transition state of the interaction for each mutant by using Eyring's plot. As shown in Fig. 3, favorable enthalpic energy change was observed for the R5-mutant, whereas the energy change for the K5-mutant was unfavorable in the transition state. These results indicate that the R5- and K5-Fab mutants bind insulin through significantly different processes and that the difference would result in distinct kinetic binding parameters for each mutant.

### Effect of charged residue introduction on Fab stability

We also analyzed the thermodynamic stability of the Fabs by DSC (Supplementary Fig. S3). The Tm value of the wild-type Fab was determined to be 76.8 °C, which is similar to typical values for other Fabs [15]. The Tm values for each mutant decreased as shown in Table 2. Moreover, during concentration for the DSC experiment, the K5-mutant became aggregated at around 0.25 mg/ml, indicating that the K5 mutation induced colloidal instability in the Fab. These results suggest that careful consideration of the mutation residues is required to avoid thermal or colloidal destabilization in association with affinity improvement. It is worth noting that the thermal stability of the R5-mutant was higher than that of the D5- and E5-mutants with strong negative electrostatic potential, while the affinity of the interaction of the R5-mutant was higher than that of the anionic mutants.

**Table 2:** T_m_ value of Fabs.

**Fab**	**T**_**m**_**(°C)**
Wild type	76.8
R3-mutant	74.4
R5-mutant	71.1
K5-mutant	N.D.
D5-mutant	66.9
E5-mutant	69.5

## Discussion

In this study, we introduced charged amino acid residues into the framework region of an anti-insulin Fab and investigated the effects on the interactions between the Fabs and insulin. Our results showed that introduction of basic amino acids improved the affinity. The results showing that the introduction of both arginine (R) and lysine (K) residues each improved affinity suggest that the affinity enhancement is likely to be due at least partly to the positive charge from those residues. On the other hand, the differences in the thermodynamic parameters for the Fab–antigen interactions in the transition state suggest that the underlying molecular mechanism by which introduction of either the arginine or lysine residues increases the affinity is not identical. Given that the mutation sites are located close to the CDR-L2 loop and that the balance between *k*_on_ and *k*_off_ values for each mutant is different, the mutation might affect the structural arrangement of the CDR loop in the encounter complex.

The net charge of most proteins distributes from − 5–5 [16]. Insulin is a small α-helix protein composed of 110 amino acids with 17 charged amino acid residues; the net charge of the whole insulin protein is − 3 at pH 7.4, making insulin antigen electrostatically comparable to most proteins in character. Thus, the approach of this study could be applied to a variety of antibodies. With conventional affinity maturation strategies, such as directed evolution, which often attempt affinity enhancement by improving the shape complementarities of the paratopes and epitopes [17], [18], it is essential to identify structural data and / or hot-spots. In contrast, because our strategy does not involve shape complementarities, precise characterization of the Fab and its antigen is not required. In addition, while there is a tradeoff between affinity and thermal stability in many cases [19], [20], our result showed that the Tm value of the R5-mutant, which have strongest affinity among the mutants generated in this study, was higher than D5- or E5-mutants, implying that R5-mutation might have a potential to overcome the tradeoff. On the other hand, the K5-mutation, another positive charge residue introduction, resulted in colloidal destabilization. Taken together with the SPR result showing that K5-mutant showed highest k_on_ value, the colloidal destabilization of the K5-mutant might destabilize monomer state and thereby accomplish faster association. The combination of other techniques such as incorporation of disulfide bridge into FR3 region [21] may improve the thermal and / or colloidal stability of the mutants and allow to produce a Fab that have decent stability as well as high affinity.

Although further studies using additional antibodies for other antigens would be required to validate the generality of our strategy and further optimization is needed, our strategy could be a versatile and simple means to improve the affinity of a Fab to its antigen. Additional studies that apply our strategy to antibodies that recognize highly charged antigens will provide additional insights into the underlying molecular mechanism and may extend the potential of the strategy.

## Appendix A Supplementary material

Supplementary Figure S1**. Thermodynamic parameters governing binding of Fabs to insulin**. Thermodynamic analyses of each Fab were performed at five temperature points (283.15 K, 288.15 K, 293.15 K, 298.15 K, and 303.15 K). The standard state Gibbs energy change upon binding (∆G) was obtained from Equation 1: ∆G = R T ln K_d_ (Eq. 1) where K_d_ is the dissociation constant, expressed in units of mol.l^−1^, R is the gas constant, and T is absolute temperature. The ∆G values of each data set were plotted against the temperatures, and were fitted with the nonlinear van’t Hoff equation (Equation 2), ∆G = ∆H − T∆S + ∆Cp (T − 293.15) − ∆Cp T ln (T/293.15) (Eq. 2) where ∆H and ∆S are, respectively, the binding enthalpy change and entropy change at 293.15 K, and ∆Cp is the heat capacity change, which is assumed to be temperature independent. Supplementary Figure S2**. Fab reactivity to insulin in different concentrations of sodium chloride**. The ELISA assay in the presence of different concentrations of NaCl to evaluate the contribution of electrostatic interaction in the interaction for E5- and R5-mutants. The absorbance at 450 nm was normalized using the value in the absence of NaCl. Supplementary Figure S3**. DSC analysis of each mutant.** The concentrations of the proteins were 0.25 mg/ml in PBS (pH 7.4). Heating was at 1 °C/min, and the scanning was performed from 30 °C to 90 °C. The data were normalized by subtracting the response for PBS alone from the experimental responses measured. Supplementary material.
