# Supplementary material for: Improvement of antibody affinity by introduction of basic amino acid residues into the framework region
Source: Biochem Biophys Rep. 2018 Jul 14;15:81–5. doi: 10.1016/j.bbrep.2018.07.005 (PMC6068084; doi:10.1016/j.bbrep.2018.07.005)
Supplement: Supplementary file 1 — Supplementary Figure S1. Thermodynamic parameters governing binding of Fabs to insulin. Thermodynamic analyses of each Fab were performed at five temperature points (283.15 K, 288.15 K, 293.15 K, 298.15 K, and 303.15 K). The standard state Gibbs energy change upon binding (∆G) was obtained from Equation 1: ∆G = R T ln Kd (Eq. 1) where Kd is the dissociation constant, expressed in units of mol.l−1, R is the gas constant, and T is absolute temperature. The ∆G values of each data set were plotted against the temperatures, and were fitted with the nonlinear van’t Hoff equation (Equation 2), ∆G = ∆H − T∆S + ∆Cp (T − 293.15) − ∆Cp T ln (T/293.15) (Eq. 2) where ∆H and ∆S are, respectively, the binding enthalpy change and entropy change at 293.15 K, and ∆Cp is the heat capacity change, which is assumed to be temperature independent. Supplementary Figure S2. Fab reactivity to insulin in different concentrations of sodium chloride. The ELISA assay in the presence of different concentrations of NaCl to evaluate the contribution of electrostatic interaction in the interaction for E5- and R5-mutants. The absorbance at 450 nm was normalized using the value in the absence of NaCl. Supplementary Figure S3. DSC analysis of each mutant. The concentrations of the proteins were 0.25 mg/ml in PBS (pH 7.4). Heating was at 1 °C/min, and the scanning was performed from 30 °C to 90 °C. The data were normalized by subtracting the response for PBS alone from the experimental responses measured. Supplementary material. [file mmc1.pptx]

## Slide 1
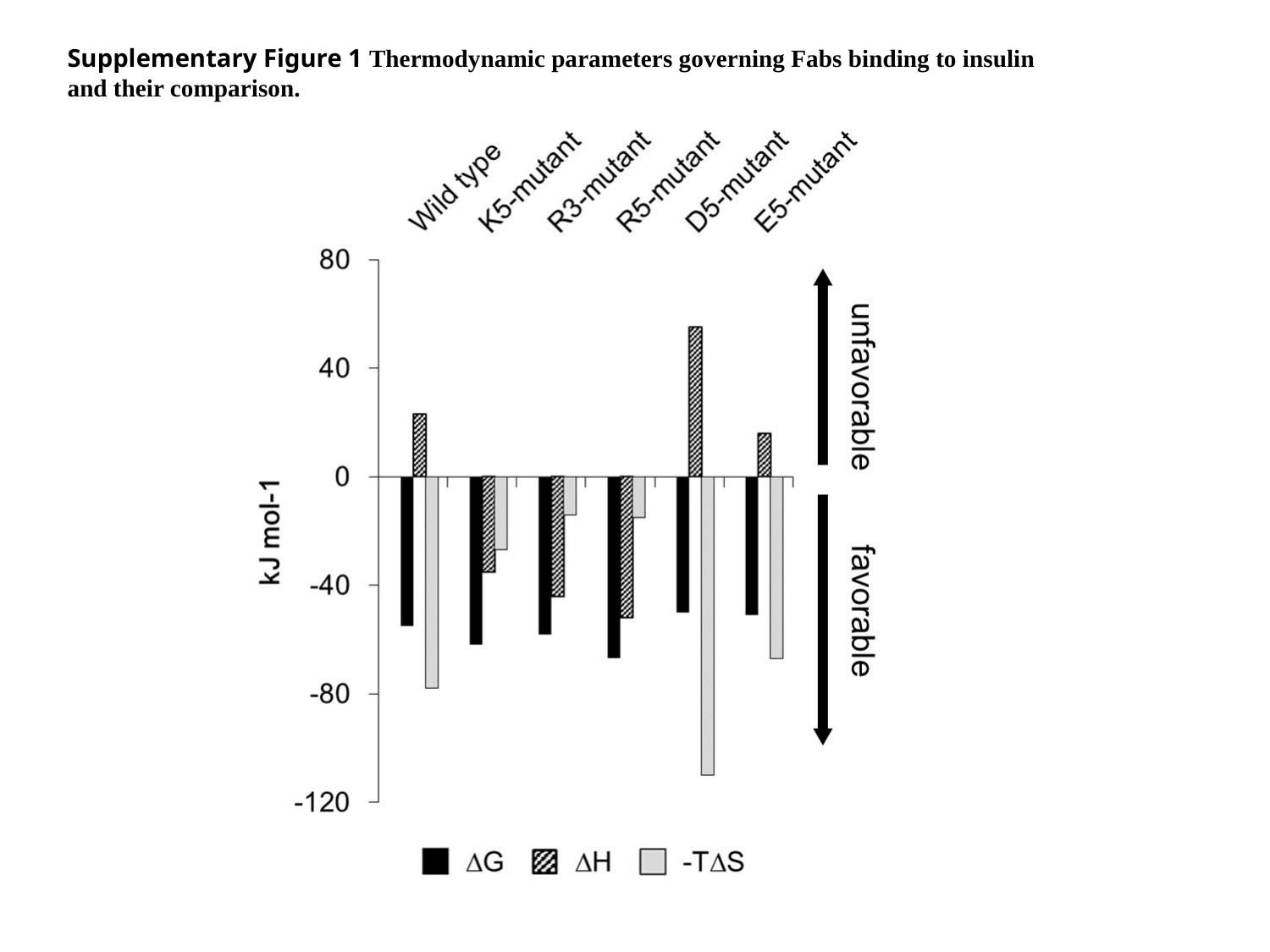

Supplementary Figure 1 Thermodynamic parameters governing Fabs binding to insulin and their comparison.

## Slide 2
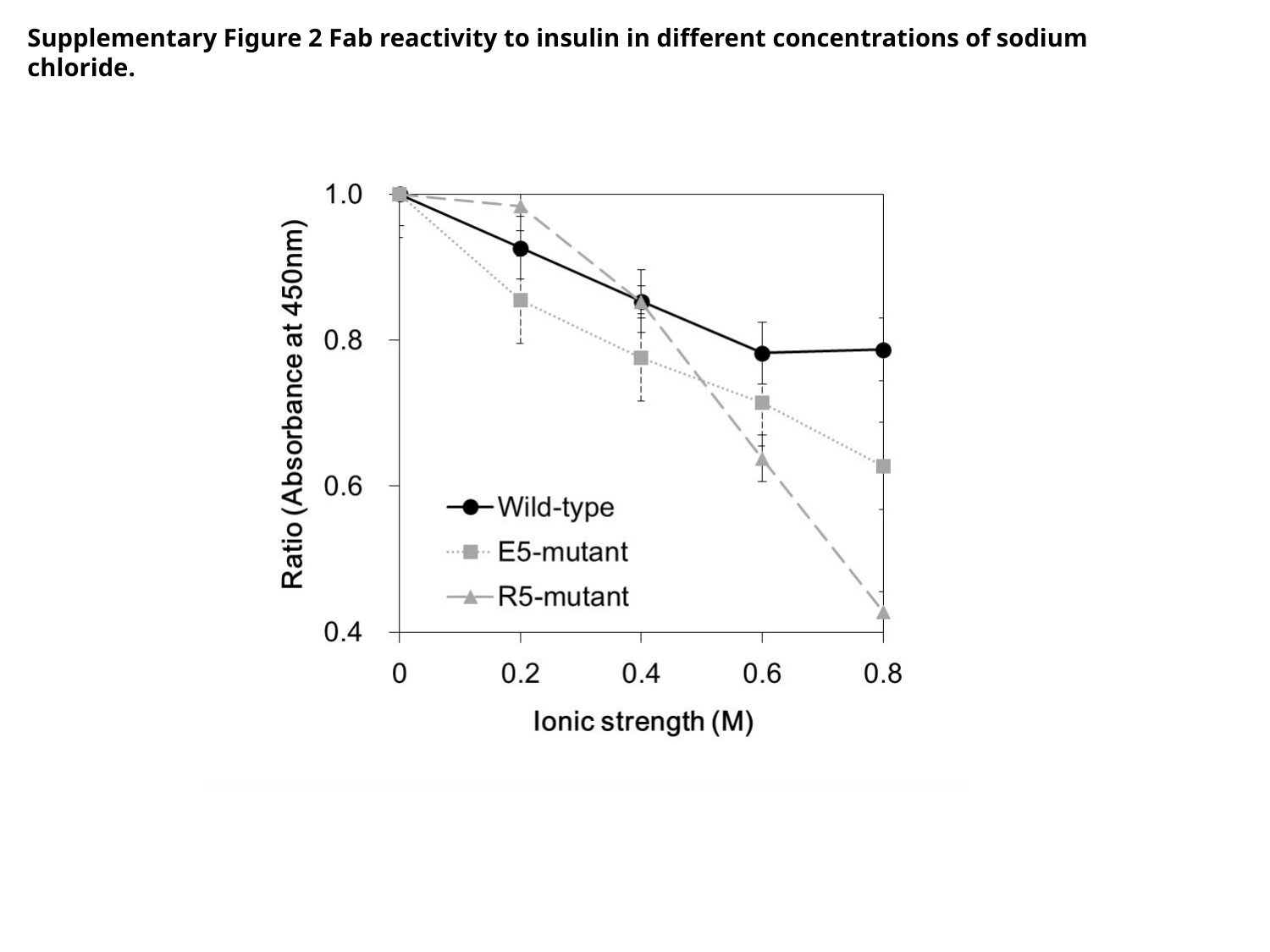

Supplementary Figure 2 Fab reactivity to insulin in different concentrations of sodium chloride.

## Slide 3
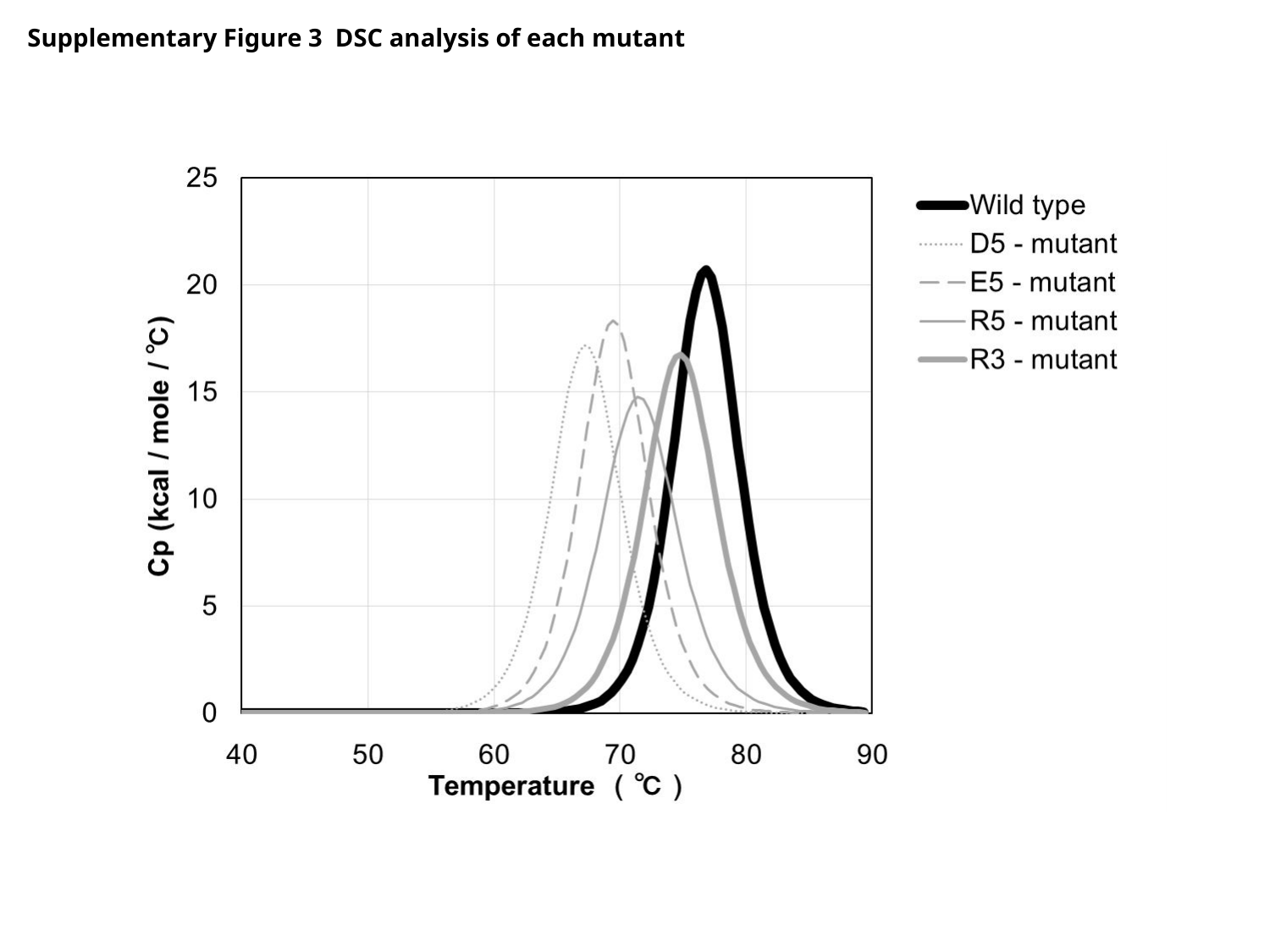

Supplementary Figure 3 DSC analysis of each mutant
